# Fast Phenomics in Vineyards: Development of GRover, the Grapevine Rover, and LiDAR for Assessing Grapevine Traits in the Field

**DOI:** 10.3390/s18092924

**Published:** 2018-09-03

**Authors:** Matthew H. Siebers, Everard J. Edwards, Jose A. Jimenez-Berni, Mark R. Thomas, Michael Salim, Rob R. Walker

**Affiliations:** 1 CSIRO Agriculture and Food, Waite Campus, Urrbrae 5064, Adelaide, Australia; m.h.siebers@gmail.com (M.H.S.); everard.edwards@csiro.au (E.J.E.); mark.r.thomas@csiro.au (M.R.T.); 2 High Resolution Plant Phenomics Centre (HRPPC), Australian Plant Phenomics Facility (APPF), Cnr Clunies Ross St and Barry Dr, Acton 2601, Canberra, Australia; berni@ias.csic.es (J.A.J.-B.); michael.salim@csiro.au (M.S.); 3 Instituto de Agricultura Sostenible (IAS), Consejo Superior de Investigaciones Científicas (CSIC), 14004 Córdoba, Spain; 4 CSIRO Agriculture and Food, CSIRO Black Mountain Science and Innovation Park, Cnr Clunies Ross St and Barry Dr, Acton 2601, Canberra, Australia

**Keywords:** phenomics, light detection and ranging (LiDAR), grapevine, proximal sensing

## Abstract

This paper introduces GRover (the grapevine rover), an adaptable mobile platform for the deployment and testing of proximal imaging sensors in vineyards for the non-destructive assessment of trunk and cordon volume and pruning weight. A SICK LMS-400 light detection and ranging (LiDAR) radar mounted on GRover was capable of producing precise (±3 mm) 3D point clouds of vine rows. Vineyard scans of the grapevine variety Shiraz grown under different management systems at two separate locations have demonstrated that GRover is able to successfully reproduce a variety of vine structures. Correlations of pruning weight and vine wood (trunk and cordon) volume with LiDAR scans have resulted in high coefficients of determination (R^2^ = 0.91 for pruning weight; 0.76 for wood volume). This is the first time that a LiDAR of this type has been extensively tested in vineyards. Its high scanning rate, eye safe laser and ability to distinguish tissue types make it an appealing option for further development to offer breeders, and potentially growers, quantified measurements of traits that otherwise would be difficult to determine.

## 1. Introduction

Phenomics is a sub-discipline of biology concerned with the rapid measurement of an organism’s phenotype or physical and biochemical make-up [1]. The ability to measure a plant’s physical and biochemical traits, the phenome, has lagged behind genomic advances. This has made it difficult to assess the performance of plant genotypes (varieties) in the large numbers required. There is a need to develop phenomic methods that can be applied in the field and in the lab to fully realize the advancements made in genetics and breeding [2,3]. Field-based phenomics is carried out on a variety of spatial scales, from proximal [4] to remote [5,6]. Recent developments in field-based buggies and hand-held technologies have focused primarily on broad acre crops [7,8,9]. These technologies have matured over the past decade, and such advances could prove useful for the development of phenomic technologies suitable for other crops, such as woody perennials. Field phenomics will move the “bottleneck” forwards from being unable to efficiently collect enough information for decision-making to dealing with the resulting large data sets and how to best utilise them. Consequently, methods of data processing and interpretation need to be developed together with the technologies for high-resolution field sensing.

In vineyards, field phenomic techniques could benefit growers both through research application and through the assessment of crop management outcomes. For example, phenomic techniques could help growers to quantify spatial variability in fruit quality, yield, vine vigour or the incidence of disease [9,10,11]. This would allow the rapid production of whole-vineyard surveys of these parameters, with multiple industry benefits, including a foreknowledge of vineyard yield. Surveying the incidence of disease or quality of grapes pre-harvest could also significantly improve the profitability of a vineyard through more efficient use of pesticides, vineyard equipment and the potential for selectively harvesting based on zones of fruit composition that would otherwise have been difficult to determine. Scientifically, proximal sensing offers tools that, individually or combined, could provide information about a plant’s canopy architecture [12,13,14,15], photosynthetic capacity [16], water status [17] or susceptibility to disease [18]. These data, together with high-speed processing, will help to address the current bottleneck of information that is needed to make informed decisions about the performance of new genotypes in comparison with existing commercial varieties in the field.

Light detection and ranging (LiDAR) is of particular interest as a tool that could be developed for use in vineyard management or phenomics [19]. LiDAR operates by firing rapid pulses of laser light at a surface. Sensors within the system measure how long it takes for the reflected light to return and its intensity. With varying degrees of precision, LiDAR sensors provide three-dimensional coordinates of reflected light. LiDAR has been successfully used as a tool to correlate plant growth in a number of ecosystems and crops [8,19,20,21,22,23]. Additionally, lasers are available at a range of wavelengths, depending on the model. In the future, multiple-wavelength LiDAR sensors could be used to detect more comprehensive physiological profiles associated with specific stages of vegetative growth [24].

Previous use of LiDAR technology in vineyards has focused on mounting relatively low scanning-rate lasers onto tractors or all-terrain-vehicles. Recently, however, researchers have developed proximal sensing vehicles that are more light-weight, easily transported between field sites and designed to allow for the easy integration of a variety of new phenotyping tools [9].

This paper introduces the frame and capabilities of an adaptable proximal sensing vehicle known as GRover (the grapevine rover). The frame and its design are a collaborative attempt to take a phenotyping platform that was successful in row-crops [9] and, after modification, apply it to grapevines. We demonstrate how GRover is able to use a high scanning-rate SICK LMS-400 red LiDAR to capture point clouds of vine size and structure at a number of different growth stages and with differing canopy management systems. The high resolution of the system and ability to co-register the individual LiDAR scans, producing 3D data, has allowed a simple but effective computational method to be used to estimate pruning weight, an indicator of vegetative vine vigour, which requires considerable labour costs to measure, as well as trunk and cordon volume. These capabilities add to previous applications of LiDAR in vineyards [13,25,26]. Further development of algorithms for processing the LiDAR point cloud will improve upon GRover’s capabilities for determining new phenotypic traits.

## 2. Materials and Methods

### 2.1. Description of the GRover’s Platform

**Figure 1:**
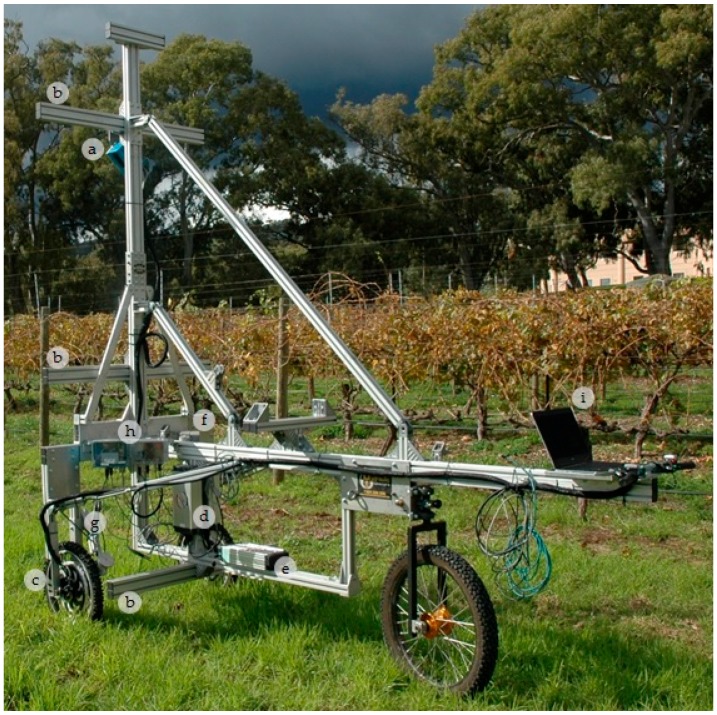
This shows GRover’s frame and components: (**a**) is the SICK LMS-400 light radar (light detection and ranging, LiDAR), mounted 2.25 m above the ground at a 45-degree angle. The LiDAR scans at 250 Hz at a wavelength of 650 nm and a range of 3 m; (**b**) shows the masts on which instruments can be mounted—each mast can be moved up, down, away from or toward the frame; (**c**) each of the front wheels contained an electric motor; (**d**) shows the 48v batteries used to power the wheels; (**e**) is the 24v instrument battery which was split into separate voltages by (**f**) the junction box; (**g**) is the spring-armed wheel encoder, which tracked the linear distance travelled during scans; (**h**) the data from the LiDAR, encoder and Global Positioning System with Inertial Measurement Unit (GPS/IMU), which was attached to the top of the LiDAR, were relayed back to (**i**) the field laptop.

The frame for GRover was completed in May 2015. It is made of lightweight structural-aluminium and weighs ~200 kg (Figure 1). It is 3 m long and has a wheelbase that can be adjusted between 1.2 and 3 m to enable operation in a variety of row spacings with maximum stability. The mast can be raised for measuring taller canopies and lowered for transportation. When raised, the mast is 3.2 m tall and stabilized by an additional aluminium support beam that is stored on the frame. GRover measures 2.1 m tall when the mast is lowered. GRover’s principle sensor, the LiDAR (SICK LMS-400, SICK Pty Ltd., Heidelberg West, Victoria, Australia) (Figure 1a) can be mounted in virtually any position on the frame. There are three cross-beams which run perpendicular to the length of GRover’s frame on which the LiDAR, or other sensors, can be mounted. Those mounts can be raised and lowered or moved toward or away from the centre of the frame (Figure 1b). The mount-points are in positions in front of and away from the frame so that line-scan sensors can be used without any interference from the frame. The LiDAR can be rotated to scan anywhere from zero degrees, pointing straight at the ground, to 90 degrees, pointed straight at the canopy, or to 180 degrees, positioned underneath the canopy, aimed skyward. For all the scans presented here, the LiDAR was mounted 2.25 m above the ground on the centre mast and angled at 45 degrees, as pictured in Figure 1.

The frame was equipped with three wheels: the front two wheels have built-in electric motors, and there was one free-pivoting wheel in the rear. The rear tire allows GRover to have a zero degree turn radius, which was important to enable manoeuvrability in looping between vineyard rows. The wheels were operated and driving speed was controlled by two thumb-throttle controllers mounted on GRover’s rear handle bars.

### 2.2. LiDAR Sensor Specifications

GRover uses an eye-safe red laser (650 nm) and scans a 70-degree field of view. The LMS-400 has a range of 0.7 m to 3 m and can be programmed to scan between speeds of 250 to 500 Hz. The LiDAR produces polar coordinates (distance and angle) from the resolved time of flight from the laser, which are then converted to *xyz* coordinates to generate a point cloud. Additionally, the LiDAR produces information about the reflectance of the scanned surface. The reflectance value is related to the ability of a material to reflect the LiDAR signal back to the sensor: the higher the reflectance value, the more reflective the surface.

The linear distance travelled by GRover was measured with an incremental rotary wheel encoder (SICK DFV60A, SICK Pty Ltd., Heidelberg West, Victoria, Australia) which provides linear travelled position. Viewed from the handlebars, the wheel encoder is in contact with the front left tire (Figure 1g). A spatial Global Positioning System with Inertial Measurement Unit (GPS/IMU) (Advanced Navigation, Sydney, Australia) was attached with double-sided tape to the top of the LiDAR (not pictured). The spatial unit is used to record data about changes in the angle of the LiDAR and GRover’s spatial position with the following specifications: GPS horizontal accuracy ±2 m, roll and pitch accuracy 0.1 degrees, and velocity accuracy 0.05 m/s. The LiDAR, GPS and encoder data are captured using the field laptop (Figure 1i) running proprietary java software developed by the High Resolution Plant Phenomics Centre (HRPPC, Canberra, Australia; http://www.plantphenomics.org.au/). The software provides a user interface presenting a map of the GPS location and input dialogs for the experiment name and run-number. The LiDAR data are stored in a custom binary format called Phenomics LiDAR Format (.PLF). The file format was developed by the HRPPC for efficient storage of LiDAR data. Detailed descriptions of the data capture software and file storage can be found in Jimenez-Berni et al., 2018 [9].

### 2.3. Scans and Voxelization

Scans made before and after pruning were used for pruning weight correlations. The scans made after pruning were used for trunk and cordon volume correlations. Scans were voxelized using the Octree function within the open-source software CloudCompare.

### 2.4. Data Processing

**Figure 2:**
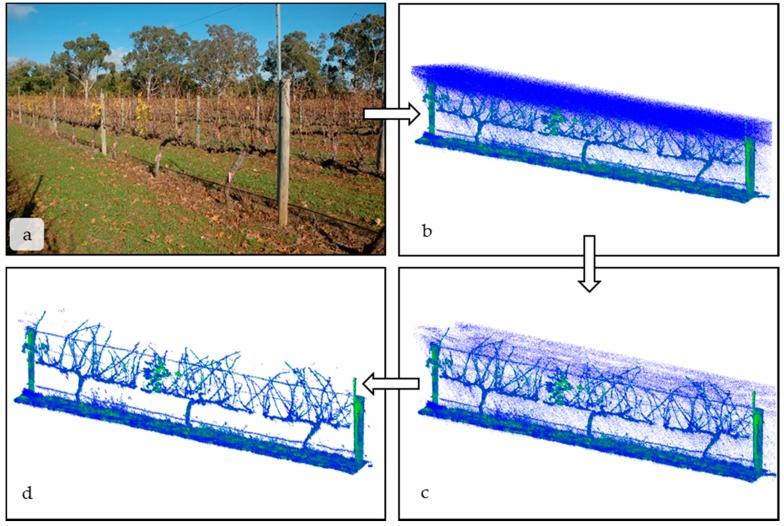
Following the arrows clockwise from panel (**a**–**d**), this figure depicts the workflow used to process scans before computational analysis. (**a**) depicts a single panel of vines in May 2015 after nearly complete senescence; (**b**) shows the raw LiDAR scan of the panel before any erroneous points are removed using an intensity filter. Less reflective, low intensity returns are blue (e.g., green grass) and more reflective, high intensity points (e.g., senesced leaves, leaf litter) are green and yellow; (**c**) shows the LiDAR scan after removal of low intensity points; (**d**) is the LiDAR scan after a nearest neighbor filter is applied to remove any outliers. All scans are visualized and processed using the open source program CloudCompare.

Custom software was developed in Java programming language (Oracle, https://www.oracle.com/java/index.html) to convert the .PLF and GPS data to a standard point cloud format, the Stanford triangle format (.PLY). At present, the GPS data is used only to position the point cloud as a whole in 3D space, but could potentially be combined with the LiDAR and encoder data in the future. The integrated point cloud and encoder data, saved as a .PLY file, were visualized using CloudCompare [27]. Point clouds were processed and cleaned using two applications (Figure 2a–d). First, the points were filtered by their reflective intensity using an intensity selection plugin built into CloudCompare (Figure 2b,c). All points with reflective intensities less than or equal to 1 were removed to minimise spurious returns (see also Section 3.2 below). Second, the Point Cloud Library (PCL) wrapper plugin was used, which employed a statistical outlier removal filter [28] based on a nearest-neighbour filtering algorithm (Figure 2c,d). In detail, 10 points were used for mean point distance estimation and the standard deviation multiplier threshold was 1.00. The work flow, rationale for choosing specific thresholds and need for point cloud pre-processing are provided in Section 3.2, the results and discussion.

### 2.5. Vineyards

The vineyards used in the study were at two separate locations:

- Alverstoke teaching vineyard, University of Adelaide, Waite Campus, Adelaide, South Australia. Variety Shiraz, trained to a single cordon and spur or minimally pruned. A single foliage wire was used to vertically shoot position the vines during the growing season. This has a mildly sloping terrain with a winter mid-row cover crop;
- South Australian Research and Development Institute (SARDI) research vineyard at Nuriootpa, South Australia. Variety Shiraz, trained to a single cordon and spur pruned. Vines were allowed to ”sprawl” during the growing season. Flat terrain. One planting of the Shiraz variety at this location was used for comparison of LiDAR scans with cordon and trunk volume measurements and another used for comparison with pruning weight measurements. Measurements were made in winter 2015.

## 3. Results and Discussion

### 3.1. Prototype Testing of Platform Robustness and Scanning Speed

Platform robustness: Testing at a range of sites demonstrated that GRover is capable of being wheeled onto a trailer by one person, anchored and ready for transport in ~30 min. The ease of transport gives the platform an advantage over some previous phenotyping platforms which are more cumbersome and difficult to transport. For example, some earlier phenotyping platforms are larger tractor-like assemblies [29], stationary towers [30] or larger buggy-like vehicles [8]. Minor shortcomings of the platform design were identified during initial testing and rectified; for example, the folding mast hinge was easily damaged due to twisting during transport, which was solved using additional transport fittings.

Scanning speed: Initial testing at the University of Adelaide teaching vineyard highlighted the importance of scanning speed. GRover was driven at an average speed of 2 km/h. Given that both sides of the vine had to be scanned separately, a one km vineyard-row was able to be scanned every hour. These scanning speeds lie within the range of what has previously been reported in other LiDAR based studies. For example, tractors or all-terrain vehicles equipped with another type of SICK laser, the LMS-200, travelled between 1 and 4.5 km/h [31,32].

Slower speeds and denser scans resulted in more effective post-processing and filtering of data. Additionally, the density of the point cloud affects one’s ability to correlate LiDAR scans to biological parameters. A conclusion from previous work using a different LiDAR to the one used in this study for measuring canopy density and leaf area in vineyards was that “scanner actuation”—or the speed at which the laser could operate—was one of the biggest issues affecting the quality of the results obtained [25]. Indeed, work in other crops reaffirms that the fast scanning rate of the LMS-400 is beneficial for volume-based determinations of biomass [33].

### 3.2. Workflow Protocol Refinement: Plot Selection and Scan Cleaning Using Two Filters

**Figure 3:**
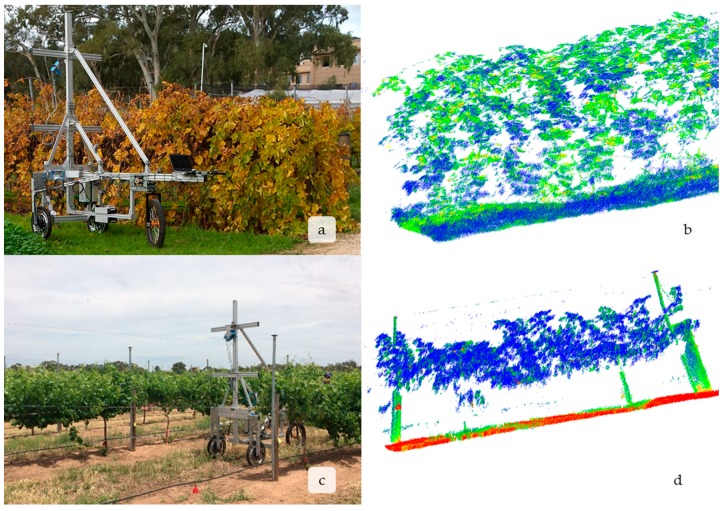
This demonstrates GRover’s ability through the LMS-400 to capture diverse features of vine growth. This includes minimally pruned vines (**a**,**b**) and spur-pruned vines trained to a two-wire vertical trellis (**c**,**d**); right panels are the LiDAR scans that were produced from the use of GRover. Pictures of GRover in the field taking the scans are to the left. All scans are shown after they were pre-processed according to the steps outlined in Figure 2. Both panels show the 3D point cloud produced and relative intensity of each point from the scan. Note how the intensity of the LiDAR return allows for visual distinctions between wood, leaf and even leaves at different phenological stages. The blue points, green leaf tissue (**d**), have the lowest intensity values and are the least reflected. Active, photosynthetic leaf tissue absorbs much more red light (**d**) than older senescent, yellow leaves (**c**) or woody tissue (**b**,**d**) which appear green (**b**,**d**). The brown soil and the identification tags on the posts in 3d are the most reflective objects and appear red.

The first step in the workflow was choosing an area to scan. Because of the adjustable wheel base, GRover could be used in almost any commercial vineyard. Vines in the single panel depicted in Figure 3a were the variety Shiraz grown at the University of Adelaide teaching vineyard. The smallest units identified for scans were single ~3.6 m panels (three vines per panel, with spacing between vines of 1.8 m) and the largest area scanned, to date, was a 500 m row containing 93 similarly sized panels (data not shown). The 500 m row produced ~1 GB. LY files for visualization. Individual plots used for collecting ground-truth data (e.g., a vine panel) were manually cut from the parent data set using CloudCompare to ensure all data were obtained from the same area of the vineyard.

After a scan was saved and visualized in CloudCompare, the point cloud was cleared of erroneous data points (Figure 2a,d). There were three main sources of error when collecting scans:

(1) Encoder dislodgement: Debris caught in the wheel sometimes dislodged the encoder, stopping it from spinning and tracking distance. This compressed the scan into two dimensions. There is no easy computational way of solving this problem, so the encoder was monitored during scans;
(2) Light intensity: Although the LMS-400 gives precise spatial and reflectance data at a high rate, it is designed to operate below 2000 Lx and not under high light conditions. Lx values in indirect sunlight commonly range between 1000 Lx on an overcast day to 130,000 Lx in direct sunlight [3]. High light levels are the cause of the spurious, low-intensity blue points seen between the LiDAR and the vines in Figure 2b. However, the erroneous measurements are all low reflectance values and can be removed by filtering the scan based on a set reflectance value. Points with reflectance values ≤1.0 were removed from the scan using the “filter points by value” plugin in CloudCompare (Figure 2b,c). In Figure 2b, there are 1.49 million total points, and 28% of those points were ≤1.0. The reflectance threshold was chosen qualitatively and removed spurious points without significantly affecting the biological interpretation of the scan. Green leaf material had a reflectance value between one and five;
(3) Edge scattering: As with any LiDAR scan, there was scattering at the edges of objects, where light is reflected in unpredictable ways. An example of edge scattering can be seen between the main vine cordon in Figure 2b. The nearest neighbor statistical outlier plugin in CloudCompare [27] removed sparse outliers based on the distance of an individual point from its neighbors. By applying these filters, point-clouds were reduced to only the scanned objects. In Figure 2c, there are 1.12 million points. After the filter removing statistical outliers is applied, there are 1.04 million points in the final point cloud (Figure 2d) on which any computational analysis would be performed.

### 3.3. LiDAR is Able to Capture Vine Size and Structure at All Growth Stages

Vines differ remarkably in size, age, management style and variety, which ultimately affects fruit quality and productivity [34]. Thus, it was important to test the practical limitations of LiDAR on a variety of vines and growth-stages. Once a consistent point-cloud pre-processing workflow was established, GRover was used to scan a diversity of vine growth throughout 2015 growing season.

**Figure 4:**
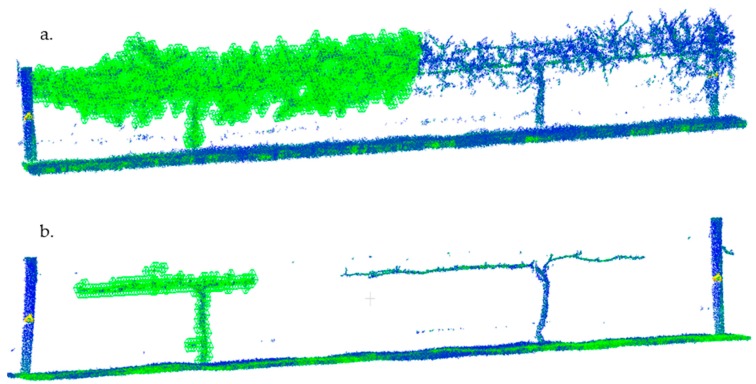
(**a**,**b**) This shows LiDAR scans made before ((**a**), *V_Before_***_(10)_**) and after cane pruning ((**b**), *V**_After_*****_(10)_**). Calculations of the voxel pruning volume (V_Diff_, Equation (1)) were made by subtracting the number of voxels (wire cubes) before (**a**) and after (**b**) pruning and comparing that value to the actual, physical pruning weight. The voxel number of post-pruned vines (**b**) was used to correlate LiDAR scans with trunk and cordon volume. Measurements of trunk volume were calculated using callipers as described in the methods. This image also illustrates what the octree algorithm looks like when applied to a point cloud. The left vine of the two-vine panel is voxelized.

Preliminary measurements were encouraging and showed that LiDAR was able to effectively and non-destructively capture detailed vegetative data for minimally pruned canopies (Figure 3a,b) and of spur-pruned vines trained on a two-wire vertical trellis typical of many Australian vineyards (Figure 3c,d) and leafless vines in the winter (Figure 4a,b). It is worth noting that reflectance values of vine growth were distinct between different vine organs. For example, the woody stem in Figure 3d has a higher reflectance value (more reflective of the red-laser) than the green leaves. Additionally, as leaves senesce and yellow at the end of the season losing their green color, they become less absorptive (Figure 3b). Red wavelength LiDAR could potentially be used to monitor senescence or the incidence of disease in the field. There are already commercial products that measure vegetative indices, such as the Greenseeker (Trimble Agriculture, Sunnyvale, CA, USA), but a LiDAR could be used to monitor multiple traits.

### 3.4. Preliminary Computational Analysis of LiDAR Scans by Voxelization Correlate with Measurements of Shiraz Trunk and Cordon Volume and Pruning Weights

There are a number of growth—as opposed to physiological—features that vineyard managers and breeders are interested in measuring, such as vine vigour, canopy area and canopy structure. Trunk and cordon volume is a potential measure of overall vine capacity. Winter pruning wood weights are often used as a surrogate measure of vine vigour.

The Octree algorithm recursively divides the point cloud (processed using the workflow described in 3.2) into smaller and smaller subdivisions in the 3D space, creating a hierarchical tree data structure. At each tree level, the space becomes subdivided by a factor of 2 which results in eight new voxels (For more see: http://docs.pointclouds.org/trunk/group__octree.html). The voxels that contain no points are removed, leaving only those voxels with some points, which represents the volume of the scanned object. This is illustrated in Figure 4a,b, where the vines to the right have been voxelized using the octree algorithm. The green wire cubes represent the size and number of the voxels at the given level of recursion.

For pruning weight, the voxel number before pruning (Figure 4a) at recursive level “R” (*V_Before_*_(*R*)_) was subtracted from the number of voxels after pruning (*V_After_*_(*R*)_) at the same level of recursion (Figure 4b) to yield the difference (*V_Diff_*_(*R*)_):

*V_Before_*_(*R*)_ − *V_After_*_(*R*)_ = *V_Diff_*_(*R*)_ (1)

**Figure 5:**
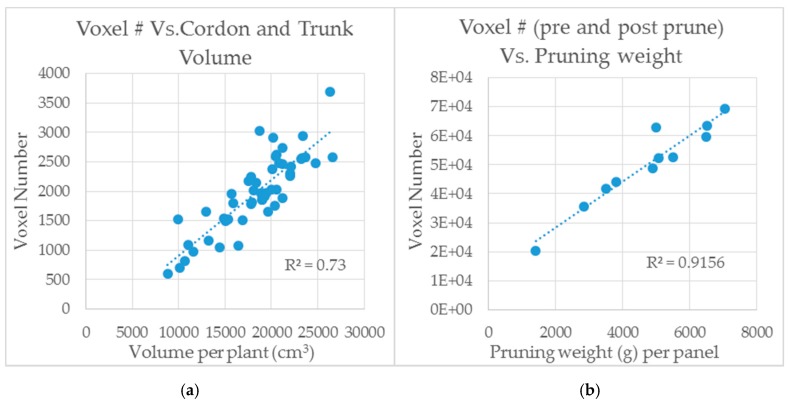
This shows the relationship between LMS-400 scans vs. trunk and cordon volume (**a**) and pruning weight (**b**). Measurements were made at the South Australian Research and Development Institute (SARDI) Research Facility, Nuriootpa, South Australia. A different planting of the Shiraz variety at the SARDI site was used for trunk and cordon volume measurements relative to that used for pruning weight measurements.

The difference between the before and after scans correlated well with destructive measures of pruning weight. At the 10th level of recursion, the R^2^ between pruning weight and scan difference was 0.92 (Table 1; Figure 5b). Cordon and trunk volume was estimated using calipers. The diameter of the trunk was measured in three even sections from its base to where the cordon split. Then diameter of each cordon, right and left side of the trunk, was measured in three places. The volume was calculated by summing the volume of each connected cylinder, from the base of the plant to the ends. Measurements of trunk and cordon volume were compared directly to the voxel number in the corresponding LiDAR scan. At the 10th level of recursion, the R^2^ between trunk and cordon volume and scan was 0.73 (Table 1; Figure 5a). At higher recursion levels, the voxels shrink to the point where they no longer simulate the object volume. Further, it is likely that higher levels of recursion, from 11 onward, began to include spurious data points not removed from filters. This would explain weaker correlations at higher levels of recursion.

Despite LiDAR being used in the past to determine pruning weight [35], that previous work was based on empirical relationships with the number of LiDAR impacts per linear meter. Our approach is based on the volumetric determination of the pruning weight as well as trunk and cordon volume using simple computational procedures, i.e., voxelization difference (Equation (1)). This has been possible due to the use of a LiDAR with a high-scan-rate and a spatial resolution (~3 mm, provided by the choice of instrument and encoder) high enough to resolve the 3D structure of the canopy and vines. It is notable that this was possible and effective using a simple, open-source algorithm. This procedure is also an improvement on previous attempts to determine canopy or wood volume and reduces the error introduced by changing LiDAR height, scanning speed or distance from the canopy [35].

Future work with GRover will involve expansion to a number of different varieties, at more locations, with a wider range of vineyard management protocols and exposure to biotic and abiotic stresses to further verify the robustness of the correlations. It will include ground-truthing LiDAR scans of vines against a number of other growth parameters, vegetative and reproductive.

## 4. Conclusions

This paper introduces GRover as a proximal sensing platform for research use in vineyards. Its frame will provide a flexible, non-destructive platform for testing multiple sensors in a variety of regions, management styles and grape varieties. It helps move the bottleneck for growers and breeders from not having enough data from vineyards to potentially having too much to analyze. This is a common problem in phenomics, but techniques for data processing and analysis are being developed together with technologies for obtaining that data, e.g., [26]. The data from GRover, once collected, is stored and can be reanalyzed as algorithms for data processing are improved, benefiting both past and current measurements. To date, the principal sensor on the frame is a relatively high-scan-rate LiDAR. The 2D line scanner has been developed to the point where it can scan one side of the vine canopy at a time and produce high-definition 3D point clouds of vine growth. The ability of the LiDAR to capture growth features has not been limited to any specific variety of vine or management style. Future work with GRover will focus on improving the algorithms for estimating growth features from the LiDAR point cloud, which will require extensive ground-truthing across multiple pruning formations and canopy architectures. Additional effort will be necessary to automate the data processing and minimise human intervention, which includes autonomous navigation of GRover. However, the motorized front wheels of Grover, the inclusion of Real Time Kinematic (RTK) GPS navigation with centimetric accuracy and the open-row nature of vineyards are all factors that suggest automation would be possible. We also foresee incorporating other types of sensors, such as stereo-RGB cameras, hyperspectral scanners or infrared thermography cameras, which will contribute to determining novel physiological traits of interest for breeders and growers, such as yield forecasting, which has attracted significant effort over the last decades [36,37,38,39,40].

## Figures and Tables

**Table 1:** This shows the R^2^ of the linear correlation between the voxel number, pruning weight and trunk and cordon volume at increasing levels of octree voxelization (recursion level _(R)_). Levels of recursion range from 6 to 11. The highest R^2^ value for pruning weight was recursion level 10 and for trunk and cordon volume it was recursion levels 9 and 10.

Recursion Level _(R)_	6	7	8	9	10	11
R^2^ (Pruning weight)	0.088	0.47	0.71	0.86	0.92	0.78
R^2^ (Trunk & Cordon volume)	0.25	0.59	0.65	0.73	0.73	0.72
